# Increasing Native Research Leadership Through an Early Career Development Program

**DOI:** 10.3389/fpubh.2022.770498

**Published:** 2022-02-24

**Authors:** Jerreed D. Ivanich, Michelle Sarche, Evan J. White, Sarah Momilani Marshall, Helen Russette, Jessica Saniguq Ullrich, Nancy Rumbaugh Whitesell

**Affiliations:** ^1^Centers for American Indian and Alaska Native Health, Colorado School of Public Health, University of Colorado Anschutz Medical Campus, Aurora, CO, United States; ^2^Laureate Institute for Brain Research, Tulsa, OK, United States; ^3^School of Social Work, College of Health and Society, Hawaii Pacific University, Honolulu, HI, United States; ^4^Center for American Indian Health, Department of International Health, Johns Hopkins Bloomberg School of Public Health, Baltimore, MD, United States; ^5^School of Social Work, University of Alaska Anchorage, Anchorage, AK, United States

**Keywords:** American Indian, Alaska Native, health equity, scholarship, early career academic, Native Hawaiian

## Abstract

Inequities impact American Indian, Alaska Native, and Native Hawaiian populations across various health conditions; in particular, many Native communities bear a disproportionate burden of substance use disorder. Such inequities persist despite concerted efforts of communities and significant research directed toward prevention and intervention. One factor hampering these efforts is the underrepresentation of researchers who are themselves Native and uniquely equipped to respond to the needs of their communities. This paper describes the innovative Native Children's Research Exchange (NCRE) Scholars program, now entering its ninth year of successful career development support for emerging Native scholars. We summarize the history of NCRE Scholars, outline the mentoring and training approaches taken to meet the unique needs of early-career Native scholars, and present key progress of program alumni. The current cohort of Scholars provide first-person perspectives on how four key program elements have supported their career development to date. NCRE Scholars has been an effective approach for supporting the next generation of Native research leaders and for helping to build an essential mass of Native researchers prepared to respond to Native community health priority needs.

## Introduction

Two facts about American Indian, Alaska Native, and Native Hawaiian (i.e., Native) populations and substance use stand in striking opposition. First, statistics document problematic patterns of substance use and rates of disorder that have remained stubbornly high despite concerted efforts by both Native communities and academic researchers to develop effective prevention and intervention strategies. Disproportionate rates of substance use disorder in Native communities are well documented (1–4). Research suggests that Native youth are at heightened risk for early substance use and the development of substance disorder relative to their non-Native peers (5–10), and that the opioid crisis has exacerbated this inequity (11–15). These realities are discouraging, particularly given extensive efforts to address them by Native communities, researchers, and funders alike.

In contrast to these data documenting the tremendous need for solutions to substance use and disorder in Native communities are those that demonstrate notable underrepresentation of Native researchers who serve as Principal Investigators (PIs) of National Institutes of Health (NIH) grants addressing these priorities. Recent efforts by the NIH, including the establishment of the Intervention Research to Improve Native American Health (IRINAH) Initiative in 2012 and the Tribal Health Research Office in 2015, have been instrumental in addressing this underrepresentation. Nonetheless, as a group, Natives have been the most significantly underrepresented group among NIH PIs (16, 17) and the NIH continues to highlight the underrepresentation of Native scientists funded through extramural research (18).

This gap in Native leadership of research related to substance use and disorder is likely hampering innovative breakthroughs to disrupt inequities (19). Although culturally sensitive non-Native researchers can effectively lead research with Native communities, provided they are responsive to community needs and priorities, we believe that Native researchers will ultimately be more effective. Numerous non-Native PIs who work closely with Native communities, effectively employing community-based participatory research approaches to enhance the rigor and relevance of research to address health inequities (including a co-author of this paper, Whitesell, who has collaborated with Native communities for 20 years). Nonetheless, Native researchers–including co-authors Sarche, Marshall, Russette, Ullrich, White, and Ivanich–offer critical perspectives from their lived experiences of *being* Native (17). Such investigators have the potential to deeply integrate rigorous scientific approaches and cultural knowledge throughout study design, measurement, analysis, interpretation, and dissemination in ways that non-Native PIs cannot, and are thus uniquely positioned to play leadership roles in research that will generate scientific breakthroughs needed to achieve health equity for Native communities.

### Strategic Mentoring of Native Researchers

Closing the gap in NIH PI representation will rely on intentional efforts that go beyond traditional training and mentoring programs for students and early career researchers. An approach focused explicitly on the needs of diverse Native scholars is indicated. Two elements are critical in this regard. First, we must identify and engage with Native graduate students, post-doctoral trainees, and junior faculty dispersed across the country, often working in or near their home communities and frequently in relative isolation from other Native researchers. Participants in the last three cohorts of NCRE Scholars, for example, are from Alaska, Arizona, Hawaii, Oklahoma, Oregon, Michigan, Montana, Washington, and Wisconsin. Bringing these investigators together is both challenging and essential, and is a feature of the program many mentees have highlighted as fundamental to their successful career development. Connecting Scholars to other Native investigators–both as mentors and peers–is vital.

The second critical element is providing mentorship, support, and training tailored to the unique needs, cultures, contexts, and goals of Native researchers.^1^ The number of Native researchers is both small and diverse. Those poised to benefit from early-career mentoring at any particular time is fewer still and includes a range of graduate students, post-docs, or junior faculty from diverse Native backgrounds and communities. The NCRE Scholars whose perspectives are shared in this paper are from five different Native communities: *Chippewa-Cree, Native Hawaiian*, the *Nome Eskimo Community, Tsimshian*, and the *Absentee Shawnee Tribe of Oklahoma*. Each early career researcher brings a unique perspective to this work. Early career researchers also have different visions for how they will engage with research and the community. For example, some see themselves housed within academia while partnering with communities, while others see themselves housed within their communities while partnering with academia. These different paths require different guidance and strategic planning and thus flexibility in programs designed to support the career development of Native researchers.

### The Native Children's Research Exchange Scholars Program

In 2012, the Native Children's Research Exchange (NCRE) Scholars program was created to help address the underrepresentation of Native researchers leading NIH-funded research. NCRE Scholars builds on the foundation of the NCRE network, which has provided fertile ground for growing this mentoring program. The NCRE network was founded in 2008 by co-authors Dr. Michelle Sarche (Lac Courte Oreilles Band of Ojibwe) and Dr. Nancy Whitesell. Both recognized the need to create space for researchers working with Native children and adolescents to gather to exchange information, build collaborative relationships, and mentor junior researchers. Two NCRE conferences (2008-2009) were supported by the Society for Research in Child Development Small Groups Initiative (Sarche, Spicer, and Whitesell, PIs), followed by five conferences (2010-2014) supported by the National Institute on Drug Abuse (NIDA; R13DA029391; Whitesell and Sarche, PIs), and four (2015-2019) by the Tribal Early Childhood Research Center (Administration for Children and Families, 90PH0027, Sarche, PI). In 2020, Drs. Whitesell and Sarche were awarded an R13 conference grant from NIDA (R13DA051122; Whitesell and Sarche, PIs) to host three additional NCRE conferences (2021-2023). Since the network started in 2008, NCRE has grown from 30 to more than 350 members from universities and other organizations (e.g., tribes, federal agencies, early childhood programs) around the United States and Canada. Membership includes representation from more than 40 tribes and is cross-disciplinary, including the social sciences (psychology, sociology, anthropology), medicine (psychiatry, pediatrics, dentistry, nutrition, nursing, pharmacy), public health (community health, epidemiology, global health), social work, education, and Native American Studies.

Mentorship of early-career scholars has been a priority for the NCRE network and conferences from the very beginning (e.g., providing travel support to students and recent post-graduates, connecting junior scholars with senior researchers, and highlighting the work of junior scholars with special sessions at meetings). In 2012, these efforts advanced significantly with the launch of NCRE Scholars (funded by NIDA through a series of contracts and a research education grant^2^). While NCRE itself focuses broadly on Native children's development, the NCRE Scholars program focuses more narrowly on preparing early-career scholars to lead research regarding the onset, progression, and impacts of substance use and disorder among Native children and adolescents.

Creating a mentoring program on the foundation of NCRE has had several advantages. The network has reach to support the recruitment of early career researchers, depth to provide Scholars with senior mentors, and regular opportunities for rich interaction and collaboration at national conferences. Another feature of NCRE fundamental to the mentoring program is its academic, community, and disciplinary diversity. As noted above, membership and participation in conferences include researchers embedded across academic and community settings, representing both diverse Native communities and scientific disciplines. This provides a rich context in which emerging Scholars can find mentors and engage in collaborative research as they pursue their own career pathways.

This paper reports on the NCRE Scholars Program as a model for tailoring career development support for Native researchers (and researchers from other underrepresented groups), describing the key elements of the career development approach and outcomes for the program's first nine years.

## Methods

The foundational approach we have used since the inception of the NCRE Scholars program has been to bring early-career investigators together in *small cohorts* to participate in both *Common* and *Tailored Activities* designed to leverage their previous experience and training, broaden and deepen their professional *Networks*–both with peers and senior mentors–to and help them successfully navigate transitions to subsequent phases of their careers. Here we describe each of these foundational elements.

### Small Cohorts

NCRE Scholars strategically recruits small cohorts, providing intensive mentoring to a few individuals rather than achieving greater reach with less depth. This approach has allowed us to focus narrowly on a few emerging investigators in each cohort, those whose interests align broadly with child and adolescent development within Native communities and, in particular, on the intersection of child development with substance use and disorder. Focusing on small cohorts has allowed us to tailor our mentoring plans to the particular needs of each Scholar in the program. Scholars enter the program at different points in their education and career development, come from different cultural and academic backgrounds, and have different research agendas and career goals. A singular approach to preparing them for the next step would be inefficient and ineffective. A tailored approach with large cohorts, however, would be unmanageable. Matriculating small cohorts each year has allowed us to provide the personalized attention required to truly adapt the program to be relevant to each scholar's needs and support their individual goals.

#### Common and Tailored Activities

Within each Cohort, Scholars participate in *Common* activities, providing opportunities for shared experiences, along with *Tailored* activities that support their individual trajectories.

### Common Activities

Common Activities support goals essential to all early career researchers. These include building a portfolio of publications to document expertise and productivity, writing grant applications, and developing relationships with other researchers working in the field. Common Activities have evolved over the years in response to feedback from Scholars and observations of what has been effective in supporting their progress. Here we describe critical Common Activities currently in use.

#### Intensive Writing Retreats

Intensive Writing Retreats began on an experimental basis with Cohort 4 in 2015 and were officially integrated into the program with funding for Cohort 6 in 2017. They provide opportunities for Scholars to step away from their everyday environments for protected time to focus on achieving writing goals, either toward publication of research findings or development of grant applications. During these two- to three-day retreats, Program Mentors (Drs. Sarche and Whitesell) offer feedback on drafts and analytic guidance as relevant. In addition, Scholars have opportunities for peer feedback on works-in-progress. These retreats have also include targeted technical support; for example, Dr. Whitesell created a two-day workshop on latent growth curve modeling using M*plus* for Cohort 4.

#### Virtual Writing Workshops

Scholars from across cohorts who convened at the NCRE Conference in 2017 discussed how valuable both mentor and peer-to-peer feedback had been in their writing efforts. This conversation launched Virtual Writing Workshops, monthly Zoom meetings of NCRE Scholars, past and present, that provide Scholars opportunities to strengthen their writing before submitting for journal or grant review. Each month, a Scholar shares a paper or grant application; others volunteer to serve as primary reviewers, along with either Dr. Whitesell or Dr. Sarche. All Scholars are invited to review and provide constructive critical feedback during the meeting and via written edits and comments. This forum has supported successful publication of manuscripts and submission of grant applications that have been awarded. These monthly meetings also provide regular opportunities for Scholars to check in across cohorts and across the country, fostering collaborative relationships among this generation of Native researchers.

#### NCRE Conferences

As noted earlier, the foundation of NCRE Scholars is the NCRE network of researchers. “NCRE” is included in “NCRE Scholars” because of the intimate link to the large group of experienced researchers who are poised to foster the career development of the Scholars. The NCRE conference is a central gathering space for network members, bringing together leading researchers studying an array of health factors among Native children and youth. Leading experts on substance use and disorder among Native youth regularly attend this conference, as do leading scholars working in prevention in Native communities. The conference structure involves research presentations with extended conversations afterward, rather than 5-min question and answer sessions more typical at conferences, and NCRE participants engage actively in these conversations. Scholars have the chance to see their academic heroes present (people whose articles they have read and cited) and to have one-on-one conversations with them afterward. Scholars are also required to present their research at NCRE, before senior researchers committed to supporting them by offering constructive criticism, suggestions, and support. Outside of the research sessions, NCRE conferences include many networking opportunities. Scholars have lunch and dinner with senior researchers and with their peers. They have opportunities to develop personal relationships that break down barriers and encourage them to reach out and build collaborations. They talk about ideas that inspire them in informal settings. It all works to foster connections that have blossomed into joint publications, diversity supplements, post-doctoral positions, and faculty appointments. In short, NCRE Conferences are the breeding ground for essential network connections.

#### Society for Prevention Research Conference

An additional networking and research exchange opportunity built into experiences for Scholars since the inception of the program is attendance at the annual Society for Prevention Research (SPR) conference. SPR has become a gathering place for a broad group of researchers working to address public health issues among Native populations in the United States. NIDA supports travel scholarships to SPR for graduate students and post-doctoral early career investigators; there is an active Diversity Network, and the Intervention Research to Improve Native American Health (IRINAH) Consortium frequently hosts its annual grantee meeting in conjunction with this conference. These factors contribute to the concentration of investigators attending and presenting at SPR, providing opportunities for NCRE Scholars to connect, learn, and develop collaborative relationships.

#### Didactic Trainings

In 2020, additional funding with the R25 education grant provided resources to develop additional training for NCRE Scholars and three courses were created. The first expands on the standard CITI training in the *Responsible Conduct of Research* to explore issues of particular concern and relevance to research with Native communities. Due to the COVD-19 pandemic, this course was adapted for virtual delivery, through a series of four discussion sessions with Native researcher guest discussants. Also included was the rETHICS Human Subjects Research curriculum (Research Ethics Training for Health in Indigenous Communities) (20) and *American Indian and Alaska Native Research in the Health Sciences: Critical Considerations for the Review of Research Application* (21).

The second course focuses on Grant Writing. This course was initially designed to be an intensive in-person course but was also pivoted to accommodate virtual learning due to the pandemic. The course includes an introduction to NIH institutes and centers, grant mechanisms and funding opportunities, application and review processes, structured guidance for creating a successful application (e.g., investigator readiness, realistic timelines for grant preparation, building research teams, crafting specific aims, connecting with program officials, budgeting, writing for review, obtaining feedback), and an optional session on NIH Loan Repayment Program applications. This course draws on intensive grant-writing curricula developed by advisor Dr. Spero Manson as part of the Native Investigator Development Program and the GUMSHOE program (Grantwriting Uncovered: Mentoring, Support, Help, Opportunity, and Experience), one of the NIH Common Fund's National Research Mentoring programs to increase the representation of racial and ethnic minorities among funded NIH scientists. The curricula and guest lectures have proven success in NIH funding for Native community research and understand the balance needed to push Indigenous methodologies, while appealing to NIH reviewers.

A third course focused on community-based participatory research (CBPR) was added in the spring of 2020. CBPR Chats were developed after the COVID-19 pandemic forced the cancelation of the SPR Conference, precluding a significant learning and networking opportunity generally provided to Scholars. Conversations with Scholars about their needs revealed that they were eager to hear stories of how senior researchers had established relationships with communities. CBPR Chats were organized to meet this need, providing opportunities for Scholars to meet with leading researchers to ask them about their experiences in an informal setting. The added advantage was giving Scholars a chance to get to know senior researchers, and vice versa. These chats have turned into a highlight of virtual learning during the pandemic and exemplify the value of a small cohort approach that can be flexible and responsive to the needs of the Scholars.

### Tailored Activities

In addition to the Common Activities, each Scholar creates an individual Tailored Career Development Plan (TCDP), articulating their short-term career goals–in alignment with their long-term goals–and proposing how they will use NCRE Scholars time and resources to achieve those goals. TCDP activities can include connecting with senior mentors, writing papers for publication, participating in training or workshops (e.g., statistical training, training, in particular, interviewing methods), participating in conferences, or writing grant proposals. Each Scholar is allocated funds to support the activities outlined in their TCDP, creates a budget and develops a NIH-style budget justification of each expense in relation to stated goals.

Program Mentors (Drs. Whitesell and Sarche) work with Scholars in developing TCDPs, which are then reviewed and approved by the Scholars Advisory Board; a group made up of three senior researchers (Dr. Melissa Walls, Bois Forte and Couchiching First Nation Anishinaabe; Dr. Amy West, Southern Cheyenne; and Dr. Allison Barlow) and three former NCRE Scholars (Dr. Jessica Elm, Oneida Nation, Stockbridge-Munsee Band of the Mohicans; Dr. Angela Walden, Cherokee Nation; and Dr. Jerreed Ivanich, Tsimshian, Metlakatla Indian Community). Once approved, Scholars begin working on their plans. Progress toward completion of TCDPs is evaluated through monthly meetings with Program Mentors and Scholars track their expenditure vis-à-vis their approved budgets.

## Results

The NCRE Scholars program has now supported 27 rising Native investigators in ten cohorts as they have embarked on careers related to the emergence and prevention of substance use and disorder among Native children and adolescent**s**. Twenty of these Scholars are American Indian; four are Alaska Native; two are Native Hawaiian, and one is a non-Native person of color. Fourteen were doctoral students, six postdoctoral fellows, and seven early career faculty. Some postgraduate Scholars were working in academic settings, while others were clinician researchers working in their home communities (e.g., tribal behavioral health or Indian Health Service clinics). By offering flexible mentoring, the NCRE Scholars program has supported diverse individual career pathways that we believe will be critical for a research workforce prepared to work collaboratively with tribal communities to create equity around substance use and disorder for Native communities.

Quantitative metrics document the progress of NCRE Scholars. Of 19 Scholars across eight Cohorts who have completed the program to date, 15 have obtained academic promotions since beginning the program (see Table 1). Nine have been promoted to Assistant Professor (or the equivalent, e.g., Assistant Scientist) and one was further promoted to Associate Professor with Tenure. Five obtained post-doctoral fellowships after being graduate student NCRE Scholars. Three have moved into community-based positions, working as epidemiologists, clinicians, or program directors in tribal communities; one took a position with the Centers for Disease Control and Prevention. Of the eight active Scholars (Cohorts 9 and 10), four are currently still in doctoral training, one has obtained a postdoctoral fellowship, and the remaining three are Assistant Professors (one promoted as a Scholar).

**Table 1:** Metrics of the progress of NCRE Scholars in Cohorts 1-10.

**Position at start of the program**	**Current position**	**Presentations**	**Publications**	**Grants**
**Cohort 1 | 2012**				
Clinical psychologist	Tribal vice president	31	11	3
Assistant professor	Associate professor with tenure	54	17	2
**Cohort 2 | 2013**				
Post-doctoral Fellow	Community health services Regional administrator	2	7	0
Senior researcher	Assistant professor of clinical and translational research; co-director of research and evaluation	111	65	11
**Cohort 3 | 2014**				
Student	Health service psychologist	29	3	1
Student	Assistant scientist	8	10	4
Student	Assist. professor, director of inclusion initiatives	59	15	1
**Cohort 4 | 2015**				
Post-doctoral Fellow	Tribal epidemiologist	27	12	2
Behavioral health clinic director	Assistant professor	19	4	0
**Cohort 5 | 2016**				
Student	Tribal community facilitator	1	1	2
Student	Health scientist	30	14	1
**Cohort 6 | 2017**				
Student	Assistant professor	34	25	4
Post-doctoral fellow	Assistant professor	45	16	5
**Cohort 7 | 2018**				
Post-doctoral fellow	Assistant professor	45	24	1
Student	Assistant scientist	13	14	3
**Cohort 8 | 2019**				
Adjunct faculty	Post-doctoral fellow	9	6	1
Student	Post-doctoral fellow	21	5	1
Student	Assistant professor	31	13	1
Postdoctoral research associate	Associate investigator	106	33	2
**Cohort 9 | 2020**				
Assistant professor	Assistant professor	33	9	5
Student	Intern	23	13	0
Student	Post-doctoral fellow	17	6	1
Assistant professor	Assistant professor	20	2	1
**Cohort 10 | 2021**				
Student	Student	9	5	0
Student	Student	15	3	1
Assistant professor	Assistant professor	22	10	0
Student	Student			

These metrics demonstrate that NCRE Scholars have been productive in the traditional academic sense and successful in career advancement. However, there are additional nuanced ways that the NCRE Scholars program supports these emerging researchers. As a group, Native scientists are in a unique position. As noted, they are the most underrepresented of all ethnic groups as NIH PIs; thus, they have few senior role models to follow. In addition, many are deeply embedded within cultural communities in which strong values for collective responsibility are at odds with the individualistic, achievement-based culture of academia. What it takes to get ahead in the competitive world of NIH and the need to focus on individual career development can create tension for young researchers who often have significant responsibilities as leaders in their home communities (17, 22). Mentoring Scholars thus includes supporting them as they navigate these challenges, helping them find a balance between building their own skills and being responsive to community and extended family needs. Our approach requires a focus on the long-term goals of addressing the public health needs of Native communities through science and understanding the role of prioritizing Scholars' skill building and career development in achieving that goal.

Another challenge many Native researchers encounter is that they often find themselves isolated in their work. If they are working in academic settings, they may be the only Native graduate student, post-doctoral fellow, or faculty member in the institution. Alternatively, the only person doing research with Native communities or both. These scholars may find themselves in the position of “speaking for all Natives” all too often. Scholars doing research on the ground in tribal communities, in contrast, often find themselves isolated from other researchers. There are challenges in finding a balance between pressing needs for service in the community and doing research in academic settings. These scholars may find themselves in the position of “speaking for all researchers.” One of the things NCRE Scholars have told us that is of most value about the NCRE Scholars program is that it brings them out of isolation and connects them with other Native researchers. We believe that the bridges built among these Scholars do as much for their career development as the discrete training opportunities the program supports. The relational bridges *sustain* them moving forward. The impact of these “soft supports” is hard to quantify, but feedback from Scholars helps tell the story. Three excerpts from evaluations solicited from early cohorts, reprinted in Table 2, are particularly illustrative, and in what follows, Scholars who have recently completed the program (Cohort 8) reflect on four key elements of the program and the value of these activities for their career development.

**Table 2:** Selected evaluation responses from NCRE scholars in Cohorts 1-3.

**Cohort 1 scholar**
The experience of being an NCRE Scholar has been the most influential in my career as a researcher and continues to provide opportunities for me to grow as a professional. Thus, I will always wholeheartedly support NCRE in any and all capacities.
**Cohort 2 scholar**
Many of the techniques that I use daily in mentoring my team were learned through the two years that I spent as an NCRE scholar, and in the ongoing outreach of you both as I have developed as an independent researcher. Since my acceptance to NCRE, I have published 33 manuscripts and have received a Native American Research Centers for Health center NIH grant and an R0l award. I credit the NCRE training and nurturing in my success as an indigenous investigator. I value the ongoing support I continue to receive as a member of the NCRE network these years later.
**Cohort 3 scholar**
Becoming an NCRE Scholar was a career-changing experience for me. Before being selected for the program, I had worked with only one other American Indian researcher. I felt isolated personally and professionally; I had always been one of few or the only American Indian in every professional space I had been part of. The program provided me with resources to carry out a secondary data analysis, which enhanced my quantitative data analysis and manuscript writing skills, and paired me with two nationally recognized American Indian researchers. Dr. X*^a^* And I continue to collaborate; we have co-authored two manuscripts, and a book chapter focused on the health and experiences of American Indians. The connections I made and tremendous support I received as an NCRE Scholar directly facilitated my ability to successfully obtain pilot grant funding to carry out a research project in my own American Indian community here in Chicago. In sum, I believe this program was absolutely essential for my career development.

a *Name removed to protect the confidentiality of this Scholar*.

### Establishing a Record of Research Productivity (Dr. Evan White, Absentee Shawnee Tribe of Oklahoma)

A key factor in developing a successful academic career is strong writing capabilities and a demonstrable record of contributions to the field. This underscores the importance for Scholars to disseminate findings from our work through peer-reviewed publications, books (e.g., chapter, encyclopedia entries), and articles written for broader community consumption. NCRE Scholars has facilitated my own development in writing both manuscripts for peer-review publication and NIH grant proposals. With respect to manuscript development, the first Intensive Writing Retreat facilitated the development of an introduction section for an empirical manuscript. Specifically, within the two-day retreat, the introduction was fully drafted for the paper in preparation.

Furthermore, the monthly Virtual Writing Workshops serve as an essential review stage before journal submission. These avenues provide the opportunity for other Native scholars to offer feedback on writing, as I am the only Native Scholar at my home institution. In response to COVID-19 travel restrictions, NCRE Scholars leadership facilitated virtual writing retreats with the current cohort of Scholars *via* Zoom to ensure we could still achieve our writing goals.

Concerning grant writing, the monthly Virtual Writing Workshops enabled the solicitation of constructive critiques from a range of Native scholars on a recent proposal before its submission. As with the manuscript, this workshop is the primary avenue for me to engage with other Native scholars regarding the proposal and provides a critical perspective on the project that would otherwise be missing. For both grant and manuscript writing, future planned activities for the remainder of the program will provide immense benefit. The upcoming grant writing workshop will provide focused time and hands-on training to facilitate an NIH R21 proposal development. Also, ongoing NCRE Scholar mentoring relationships are incredibly beneficial in building collaborations for manuscripts as well as grants. As a result, I have been able to incorporate experts in American Indian research as co-authors on manuscripts as well as consultants and mentors on grant submissions. These opportunities would not have been available without NCRE Scholars' support. Finally, in addition to direct writing projects, I have received experience reviewing academic writing in a mentored setting by serving as a reviewer in the monthly Virtual Writing Workshops. This fosters skill development essential for peer review service, which is nearly ubiquitous among academic careers.

### Building Professional Connections (Dr. Sarah Momilani Marshall, Native Hawaiian)

The importance of building strong professional connections cannot be overstated. Through numerous formal and informal avenues, NCRE Scholars has provided me with crucial opportunities to develop and utilize relationships with senior mentors, early career researchers, and peers. Before my involvement with the NCRE network, I had minimal exposure to other Native researchers focused on substance abuse prevention and Native health issues. As a recent Ph.D. graduate who had chosen to remain in my rural community rather than re-locate to the city, I continuously encountered professional isolation and limited opportunities for career development. The professional connections facilitated by Drs. Whitesell and Sarche have become a vital resource for establishing relationships with other Native scientists throughout the country while remaining in my rural community. These opportunities to learn from other Native scientists have helped shape my expectations as a Native researcher, better prepared me to work with Native communities, and strengthened my identity as a Native scientist.

Another indispensable strength of NCRE Scholars is its ability to develop and maintain productive relationships among its current and former scholars and a broad national network of researchers in the field. As a Scholar in the program, I have richly benefitted from mentorship with other NCRE network researchers who have shared insights and perspectives with me from their own experience as early career Native scientists. Both peer mentors and established scientists in the field have generously shared personal challenges and triumphs and extended offers of consultation and collaboration. These unique opportunities to engage in informal conversations with peers and senior researchers have been structured around my individual professional needs and have provided me with invaluable support and guidance. Before becoming an NCRE Scholar, I could not have envisioned the depth of experience, knowledge, and wealth of resources I would receive through the program. The professional network that I have inherited as an NCRE Scholar has established a robust and critical foundation upon which I am better equipped to continue building future success.

### Strategic Planning and Support for Career Development (Helen Russette, Chippewa-Cree)

As part of the in-person orientation meeting, NCRE program mentors provided our Cohort with designated time and guidance to develop our individual Tailored Career Development Plans (TCDPs). I had selected four specific career development objectives and received a careful review from my NCRE program mentor to ensure my TCDP objectives were specific, measurable, achievable, relevant, and time-sensitive (SMART). I felt the following objectives were feasible within the NCRE Scholars period of support: submit at least one research-related manuscript as the first author to a peer-reviewed journal and submit one grant application; connect with a substantive mentor in my field of research, and develop a professional network with other Native researchers. With the ongoing support and opportunities provided by NCRE, I have made progress on several of my objectives and have completed two of them to date.

Such NCRE Scholar-supported research-related activities have helped me move forward in my research. I used a portion of our TCDP budget, which is designated solely for research-related costs, to work with another Native doctoral student to conduct an inter-rater reliability assessment for my qualitative study. My draft manuscript received a critical review from my fellow Scholars, NCRE program mentor, and members of the Scholars Advisory Board during a Virtual Writing Workshop, which allowed me to submit a more polished manuscript to a peer-review journal currently in review. I plan to submit at least one more draft manuscript to the workshop during my time as an NCRE Scholar. I, as well as another current Scholar, applied for and received diversity fellowship grants. Both of our proposals were strengthened by having letters of support from the NCRE Scholars Program Mentors. One reviewer noted specifically that a strength of my application was being an NCRE Scholar.

In light of the COVID-19 pandemic, travel has been interrupted. With support from NCRE Scholars Program Mentors, I have met and collaborated with my substantive mentor via Zoom. This career-altering connection would not be possible without NCRE Scholars. Within the first few meetings with my substantive mentor, I have gained a wealth of knowledge in my research field, which focuses on early community intervention among families with children with prenatal substance exposure. My mentor and I are currently discussing a possible post-doctoral position within their research institute upon my graduation, and I am currently collaborating on a project within their center.

### Didactic Trainings (Jessica Saniguq Ullrich, Nome Eskimo Community)

In addition to building professional connections, strategic planning, and writing productivity, my fellow NCRE Scholars and I received didactic training on community-based participatory research (CBPR) and responsible conduct of research with Native communities. Virtual workshops were held with guest speakers who have expertise in engaging Native communities in successful research. Our first guest spoke about the principles of CBPR and the importance of providing helpful research to the community. Our second guest shared what motivated them to research with Native communities and gave practical advice on the importance of “failing forward,” taking the perspectives of others, tolerating ambiguity, continuing to be reflexive, and ensuring that writing happens every day. Our third guest discussed how CBPR had made paradigmatic shifts within research more relational and community-driven because there is now a growing recognition that the community contains the knowledge and solutions. These discussions provided an opportunity to solicit practical advice for navigating the research landscape as a junior scholar, network with distinguished CBPR researchers, and learn from their collective research journeys that helped develop more effective health interventions for Native communities.

During the scheduled two-day Virtual Writing Workshop, Scholars listened to two former Scholars discuss their early career research strategies for ethically working with Native communities and how building mentoring relationships can guide community-based research. Their guidance was helpful because they could readily relate to what NCRE Scholars currently maneuver within research and academia. NCRE Scholars also had discussions with NCRE Program Mentors about the assigned readings and the conversations that took place with guest discussants, such as one Native researcher who provided her perspective on the grant review process. The next phase of the NCRE program will involve training and education about grant writing, which will be extremely valuable to junior scholars mapping out how to access funding that will benefit and serve Native communities. The training and relationships that have been established provide NCRE Scholars with connections, knowledge, and opportunities to launch successful research careers with Native communities.

## Discussion

Scientific leadership from within Native communities can play a vital role in research to inform solutions to persistent health inequities. Growing that leadership requires intentional and strategic efforts to support the development of Native students and early-career scientists. Engaging with emerging researchers in the NCRE Scholars program over the past decade, we have found four key elements critical to enhancing career progress. A focus on *establishing a record of research productivity* has helped Scholars gain practice and proficiency and document expertise necessary to obtaining jobs and grant funding. Introducing them to senior researchers, key mentors, and a network of early-career Native researcher peers enables them to *build professional connections* that generate collaboration, provides support, and reduces the sense of isolation many encounters in academic settings. Offering *strategic planning and support for career development* allows us to work with Scholars to create tailored plans for career development that honor individual goals, respect the different visions Native researchers have for working with communities to create research and adapt to varying career levels. More recently, with additional resources, we have been able to enhance Scholars' experiences through *didactic pieces of training* that allow strengthening skills and practice, particularly in grant writing, that we hope will increase their success in obtaining research funding.

The success of these strategies is reflected in the progress of Scholars across the ten cohorts to date, but perhaps most strikingly in the fact that Scholars across most cohorts remain actively connected to and committed to one another. Their strength is in the bonds they form. Many participate at least periodically in monthly Virtual Writing Workshops, and most attend NCRE conferences and convene for NCRE networking events at the annual Society for Prevention Research conference. As a testament to the strength of these connections, three past Scholars from two different cohorts submitted a collaborative NIH grant application in 2020 that was funded in 2021. As Scholar's comments above reflect, they form a network of collegial support that is an invaluable resource for many as they navigate the waters of academia–together–as Native researchers, otherwise easily drifting alone in many institutions and professional organizations. The relationships they form are meaningful, lasting, supportive, and generative. They consult with one another, share ideas, problem-solve, and together they persist and thrive.

The need for more Native researchers is clear. Native researchers can collaboratively find the solutions to the unresolved inequities within Native communities through building effective prevention and intervention strategies that will relieve the undue burden of substance use disorders. The importance of supporting the career development of emerging Native researchers is also evident because they are often thrust, too soon, into the fray. Communities and academia alike are eager for bright new Native researchers and often urge them to take up the charge too soon. They are pulled forward and sideways into commitments and responsibilities, denied the protected time and support needed to demonstrate productivity and develop research independence (23). Meanwhile, their non-Native peers have time to publish, write grants, and establish productive research careers (24).

The NCRE Scholars program is an example of how training tailored to meet the needs of Native researchers–in this case, those specifically focused on research addressing the impacts of substance use and disorder on Native children and adolescents–can have a significant and lasting impact. Programs like this are needed across scientific disciplines to support the career development of Native scientists broadly and to increase representation in the leadership of research that is with and about Native communities so that the research can be more effective in addressing the community needs and priorities. We believe that the NCRE Scholars program can be a model for successful mentorship for other underrepresented scholars in the academy.

## Data Availability Statement

The original contributions presented in the study are included in the article/supplementary material, further inquiries can be directed to the corresponding authors.

## Ethics Statement

Written informed consent was obtained from the individual(s) for the publication of any potentially identifiable images or data included in this article.

## Author Contributions

JI, MS, and NW contributed to conception, design of the study, and wrote the first draft of the manuscript. EW, SM, HR, and JU wrote sections of the manuscript. All authors contributed to manuscript revision, read, and approved the submitted version.

## Funding

This work was supported by the National Institute on Drug Abuse under Grant 5R25DA050645.

## Conflict of Interest

The authors declare that the research was conducted in the absence of any commercial or financial relationships that could be construed as a potential conflict of interest.

## Publisher's Note

All claims expressed in this article are solely those of the authors and do not necessarily represent those of their affiliated organizations, or those of the publisher, the editors and the reviewers. Any product that may be evaluated in this article, or claim that may be made by its manufacturer, is not guaranteed or endorsed by the publisher.

## Footnotes

^1^It should be noted that genuinely achieving equity in PI representation will also require engaging potential researchers earlier in the educational pipeline to expose them to research career opportunities, and we are engaged in some efforts to that end. However, those are beyond the scope of this paper.

^2^HHSN271201200692P, HHSN271201500717P, HSN271201700715P, Whitesell and Sarche, PIs; R25DA050645; Whitesell & Sarche, PIs.
